# Combined Treatment of Heterocyclic Analogues and Benznidazole upon *Trypanosoma cruzi In Vivo*

**DOI:** 10.1371/journal.pone.0022155

**Published:** 2011-07-26

**Authors:** Denise da Gama Jaén Batista, Marcos Meuser Batista, Gabriel Melo de Oliveira, Constança Carvalho Britto, Ana Carolina Mondaine Rodrigues, Chad E. Stephens, David W. Boykin, Maria de Nazaré Correia Soeiro

**Affiliations:** 1 Laboratório de Biologia Celular, Fundação Oswaldo Cruz-Instituto Oswaldo Cruz, Rio de Janeiro, Brazil; 2 Laboratório de Biologia Molecular e Doenças Endêmicas, Fundação Oswaldo Cruz-Instituto Oswaldo Cruz, Rio de Janeiro, Brazil; 3 Department of Chemistry and Physics, Augusta State University, Augusta, Georgia, United States of America; 4 Department of Chemistry, Georgia State University, Atlanta, Georgia, United States of America; Univ. Georgia, United States of America

## Abstract

Chagas disease caused by *Trypanosoma cruzi* is an important cause of mortality and morbidity in Latin America but no vaccines or safe chemotherapeutic agents are available. Combined therapy is envisioned as an ideal approach since it may enhance efficacy by acting upon different cellular targets, may reduce toxicity and minimize the risk of drug resistance. Therefore, we investigated the activity of benznidazole (Bz) in combination with the diamidine prodrug DB289 and in combination with the arylimidamide DB766 upon *T. cruzi* infection *in vivo*. The oral treatment of *T.cruzi*-infected mice with DB289 and Benznidazole (Bz) alone reduced the number of circulating parasites compared with untreated mice by about 70% and 90%, respectively. However, the combination of these two compounds decreased the parasitemia by 99% and protected against animal mortality by 100%, but without providing a parasitological cure. When Bz (p.o) was combined with DB766 (via ip route), at least a 99.5% decrease in parasitemia levels was observed. DB766+Bz also provided 100% protection against mice mortality while Bz alone provided about 87% protection. This combined therapy also reduced the tissular lesions induced by *T. cruzi* infection: Bz alone reduced GPT and CK plasma levels by about 12% and 78% compared to untreated mice group, the combination of Bz with DB766 resulted in a reduction of GPT and CK plasma levels of 56% and 91%. Cure assessment through hemocultive and PCR approaches showed that Bz did not provide a parasitological cure, however, DB766 alone or associated with Bz cured ≥13% of surviving animals.

## Introduction

Discovered by the Brazilian physician Carlos Chagas one century ago, Chagas disease (CD) is a zoonosis caused by kinetoplastid flagellated *Trypanosoma cruzi* [1]. It is well known that CD is an endemic illness in poor areas of 15 developing countries of Latin America, affecting about 12 to 14 million people. Less well known is that CD is becoming a health problem in non-endemic areas such as Europe and United States largely due to the migration of infected people to these regions [2]–[4]. Due to the lack of an efficient therapy, mainly for chronic chagasic patients, and since it has been considered by many pharmaceutical industries to have limited economical potential, CD has been designated a neglected tropical disease [5].

The main route of transmission of *T. cruzi* infection to humans is through the feces of blood-sucking triatomine insects but other routes also exist including blood transfusion, transplacentally, organ transplantation, laboratory accidents and oral ingestion of contaminated food [6]–[12]. CD is the leading cause of infectious myocarditis worldwide, which is one of its most serious and frequent clinical manifestations observed during the chronic phase of the disease that appears in about 20–40% of infected individuals years or decades after the acute infection [13]–[15]. The available treatment is based on two nitroheterocycles: nifurtimox (4-[(5-nitrofurfurylidene)-amino]-3-methylthio morpholine-1,1-dioxide), a nitrofuran produced only in El Salvador by WHO-Bayer, and benznidazole (*N*-benzyl-2-nitro-1-imidazoleacetamide), a nitroimidazole produced by LAFEPE-Brazil. Both are indicated for the treatment of all acute and early chronic cases, exhibiting about 60–80% efficacy [16]. Nevertheless, neither compound is highly effective against the late chronic phase (about 20% cures), both require long-term therapy in addition to displaying side-effects that can lead to interruption of the treatment. These deficiencies justify the search for new chemotherapeutic options [5], [17], [18], [19].

Many studies have demonstrated the excellent activity of aromatic diamidines (AD), pentamidine and related compounds, against many pathogens, such as bacteria, fungi and protozoa [20]. Although the exact mechanism of action is still not precisely known, it has been proposed that the binding of these cationic molecules in the DNA minor groove, mainly at AT-rich regions, contributes, at least in part, to their effect upon trypanosomatids [16], [21]–[22]. We have previously reported the *in vitro* and *in vivo* activity of AD and analogues such as arylimidamides (AIA) upon *T.cruzi* [23]–[26]. A recent study demonstrated that the AIA, DB766 shows superior efficacy than Bz upon different parasite strains, including those naturally resistant to Bz [27].

Combination therapy represents a promising approach for the enhancement of drug efficacy since it (i) allows the use of at least two compounds that may act upon different cellular elements and metabolic pathways (ii) may reduce drug concentrations and number of doses thus contributing to the lowering of toxic effects, and (iii) may minimize the risk of drug resistance [28]. For the reasons stated above, the combination of different trypanocidal compounds merits exploration [16], [28]–[29]. Our present goal is to evaluate *in vivo* the combined effect of Bz with the prodrug DB289 and with the arylimidamide DB766, to determine if a scheme of therapy with these drugs could reduce toxicity and improve efficacy in an animal model for *Trypanosoma cruzi*-infection.

## Materials and Methods

### Compounds

**Figure 1:**
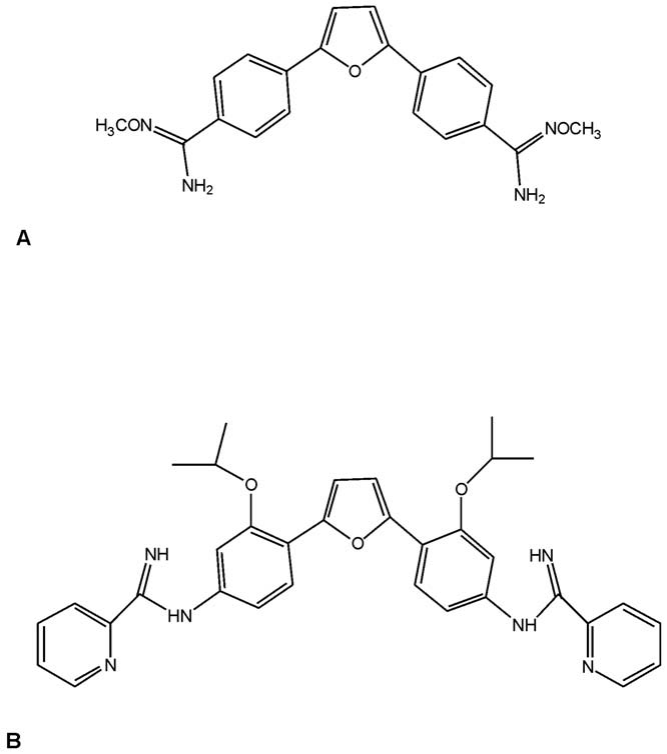
Chemical structures of DB289 (A) and DB766 (B).

The aromatic diamidine DB289 (pafuramidine maleate; 2,5-bis[4-(N-methoxyamidino)phenyl]furan monomaleate) and the arylimidamide DB766 (Fig. 1) were synthesized according to methodology previously reported by us [30]–[32]. A DB289 stock solution was made in a solvent consisting of sterile distilled water (99.4%), Tween 80 (0.1%), and ethanol, which was freshly prepared immediately before use each day. The route of administration used was oral gavage. DB289 and DB766 were dissolved in DMSO and then freshly diluted with sterile distilled water before use by intraperitoneal (ip.) or p.o. routes. The stock solution of Benznidazole (*N*-benzyl-2-nitroimidazol acetamide, Rochagan, Roche) was prepared in sterile distilled water with 3%Tween 80, and before use was diluted in sterile distilled water for p.o. administration.

### Parasites

Bloodstream trypomastigotes (BT) of the Y strain were used throughout and were harvested by heart puncture from *T. cruzi*-infected Swiss mice on the day of peak parasitemia, as described [33].

### Mice infection

Male Swiss mice (20–24 g) were obtained from the animal facilities of the Oswaldo Cruz Foundation (CECAL, Rio de Janeiro, Brazil). Mice were housed at a maximum of 8 per cage and kept in a specific pathogen free (SPF) room at 20–24°C under a 12/12 h light/dark cycle and provided with sterilized water and chow ad *libitum*. The animals were allowed to acclimate for 7 days before starting the experiments. Infection was performed by ip injection of 10^4^ BT. Age-matched non-infected mice were maintained under identical conditions.

### Treatment schemes

The animals were divided into the following groups (Table 1): uninfected (non-infected and non-treated); untreated (infected but non-treated); and treated (infected and treated with the different compounds combined or not with Bz) [24]–[25]. Drug therapy was performed by 20 daily consecutive doses (ip. and p.o., Table 1), starting at the onset of the parasitemia (5 dpi). In all assays, only mice with positive parasitemia were used in the infected groups.

**Table 1:** Cure assessment of DB289 and DB766 combined or not with benznidazole (Bz) in murine model of acute *T. cruzi*-infection.^1^

Experimental groups	Therapy route ^(^ 2 ^,^ 3 ^)^	Number of surviving/total number of animals	Assays performed after 60 days post treatment	
			Number of negative hemoculture samples/number of mice	Number of negative blood PCR samples/number of mice
Uninfected		13/13	-	-
Untreated	-	4/15	0/4	0/4
Bz 50 mg/kg/day	p.o.	14/15	11/14	0/14
DB289 25 mg/kg	p.o.	5/6	2/5	0/5
DB289 25 mg/kg+Bz 50 mg/kg	p.o.+p.o.	6/6	1/6	0/6
DB766 50 mg/kg	ip	6/15	0/6	0/6
DB766 50 mg/kg+Bz 50 mg/kg	ip+p.o.	15/15	3/15	2/15
DB766 50 mg/kg	p.o.	3/7	1/3	1/3
DB766 50 mg/kg+Bz 50 mg/kg	p.o.+p.o.	8/8	0/8	0/8

1 Swiss male mice weight 20 to 24 g inoculated with 10^4^ blood trypomastigotes (Y strain).

Treatment was initiated at 5° dpi followed by 20 daily doses.

2 Intraperitoneal – ip.

3 per oral – p.o.

### Parasitemia, mortality rates and ponderal curve analysis

The level of parasitemia was checked by the Pizzi–Brener method. Mice were individually checked by direct microscopic counting of parasites in 5 µL of blood [34]. The mortality rates were checked daily until 60 dpi and expressed as cumulative mortality (% CM). Body weight was evaluated from 0 up to 60 dpi, and expressed as percentage of weight variation [24]–[25].

### Histopathological analysis

At 14 dpi (peak of cardiac parasitism and inflammation in this experimental model as described in de Souza et al., 2006 [24], the heart tissues were removed, cut longitudinally, rinsed in ice-cold phosphate buffered saline (PBS), and fixed in Millonig-Rosman solution (10% formaldehyde in PBS). The tissues were dehydrated and embedded in paraffin. Sections (3 µm) were then stained with hematoxylin-eosin and analyzed by light microscopy. The number of amastigote nests was determined in at least 60 fields (total magnification, 40×) for each slide. The mean number of amastigotes' nests per field was obtained from at least three mice per group, with three sections from each mouse.

### Biochemical analysis

At 14 day post infection (dpi), mice blood was collected and immediately submitted to analysis for biochemical determination of plasma tissular markers including glutamate pyruvate transaminase (GPT) and total creatine kinase (CK) using the Reflotron System (Roche Diagnostics, F. Hoffmann-La Roche Ltd, Basel, Switzerland) [35].

### Cure assessment

Cure criteria were based on two parasitological methods: polymerase chain reaction (PCR) and hemoculture assays which detect kDNA minicircle specific sequences or the parasite itself, respectively. Animals presenting negative results by both tests were considered cured. Briefly, after 40 days of drug treatment, about 700 µL of blood were collected from the heart of anesthetized mice and then 500 µL and 200 µL were used for PCR and hemoculture analysis, respectively. For PCR, the blood was diluted in 1∶3 volume of guanidine solution (6 M guanidine-HCl/0.2 M EDTA), and heated for 90 s in boiling water in order to cleave the parasite kDNA network [36] and the PCR performed using the primers: (5′AAATAATGTACGGG(T/G)GAGATGCATGA3′) and (5′GGTTCGATTGGGGTTGGTGTAATATA3′), which amplify a 330 bp sequence from the kinetoplast DNA (aprox 120 000 copies per parasite), as previously described by Wincker et al. (1994) [37]. The PCR was carried out using a GeneAmp® PCR System 9700 (Applied Biosystems) as follows: one step at 94°C for 3 min (to activate the Taq DNA polymerase), 2 cycles at 98°C for 1 min and 64°C for 2 min, 38 cycles at 94°C for 1 min and 64°C for 1 min, followed by a final extension at 72°C for 10 min. The amplification products were detected on a 1.5% agarose gel electrophoresis following staining with ethidium bromide (5 mg/mL). For hemoculture, 200 µL of blood was added to 5 mL LIT medium and incubated at 28°C for 60 days, being weekly examined by light microscopy to detect epimastigote forms [38].

All procedures were carried out in accordance with the guidelines established by the FIOCRUZ Committee of Ethics for the Use of Animals (protocol approved - CEUA 0099/01).

## Results

**Figure 2:**
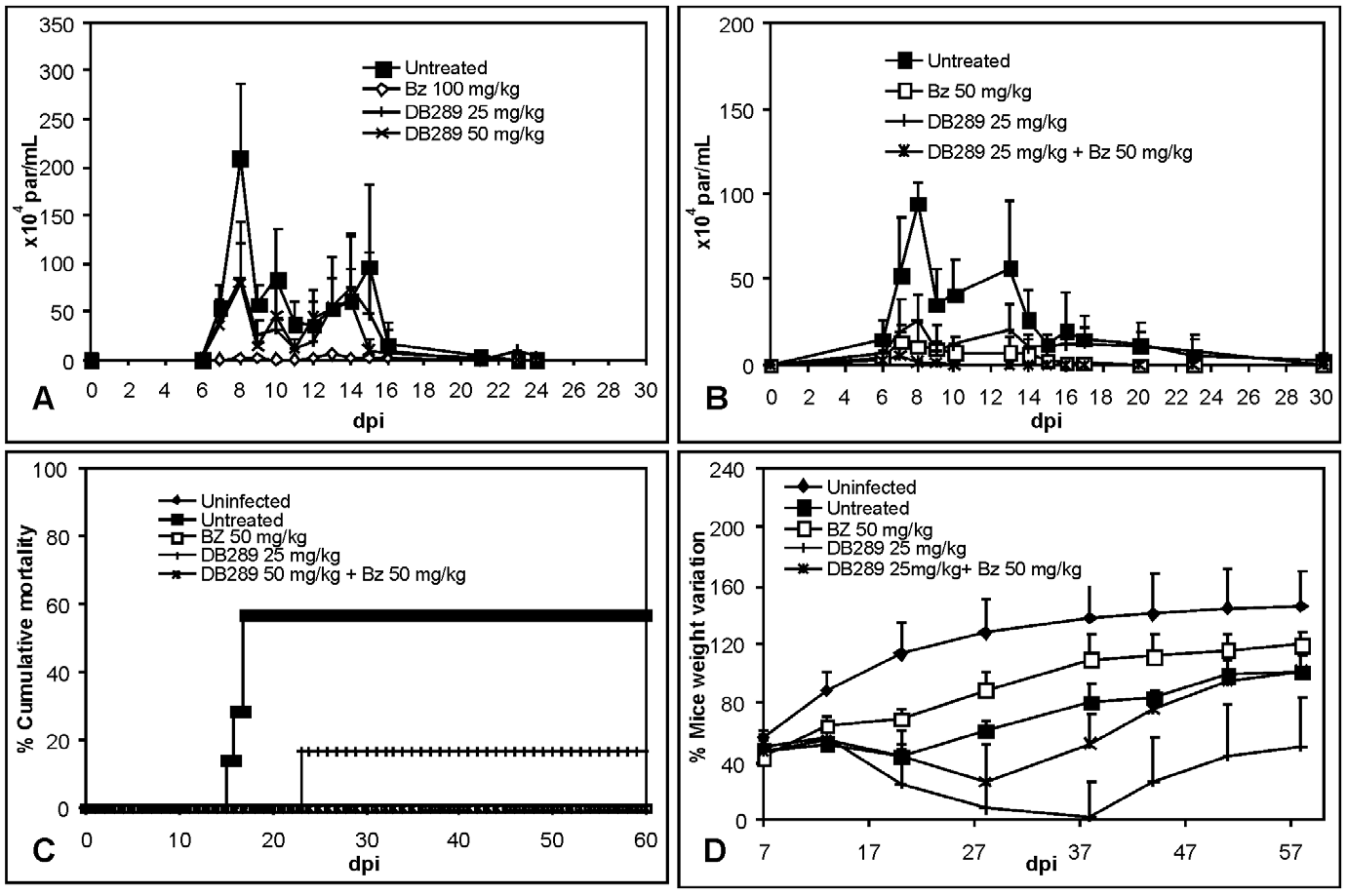
Activity of DB289 alone or in combination with benznidazole (Bz) upon *T. cruzi*-infection in mice. (A): Parasitemia curve of infected mice that were either not treated or treated at 5 and 8 dpi with 25 and 50 mg/kg/day DB289 and 100 mg/kg/day Bz. (B): Parasitemia curve, (C) Cumulative Mortality and (D) Ponderal Curve of infected mice treated or not for twenty daily doses with 25 mg/kg/day DB289 combined or not with 50 mg/kg/day.

Since (i) in a previous study we found that a phenyl-substituted analogue of furamidine gave a trypanocidal effect upon a *T. cruzi* infection *in vivo* [23], and (ii) oral administration of only two doses at onset (5 dpi) and at parasitemia peak (8 dpi) of 25 and 50 mg/kg/day of the furamidine prodrug (DB289) resulted in about a 60% decrease of parasitemia (Fig. 2A), we evaluated the combination of DB289 with Bz to determine if an enhancement of efficacy against the parasite was observed. Our results show that although treatment with 25 mg/kg/day of DB289 or 50 mg/kg/day of Bz, both alone, lowered the parasitemia peak levels by about 70% and 90%, the combined treatment reduced the number of circulating parasites at 8 dpi by more than 99% (Fig. 2B; Table 2). Mice survival rates of about 85% and 40% were found for DB289 treated and untreated mice groups, respectively. The combination of the prodrug DB289 with Bz resulted in 100% animal survival (Fig. 2C). At three weeks post infection when the highest body weight lose is observed in this *T. cruzi* experimental model [27], both DB289 alone and Bz+DB289 showed considerable lose of mice body weight similar to or even as high as that for the untreated animals (Fig. 2D). At 60 dpi, the group treated with only DB289 still showed high rates of weight loss, even more than the untreated mice group (Fig. 2D). Cure assessment evaluated by both hemoculture and PCR did not reveal a parasitological cure in any mice groups (Table 1).

**Table 2:** Parasitemia peak of *T. cruzi*-infected mice treated or not with DB289 and DB766 associated or not with Benznidazole (Bz).

Experimental group	Therapy route	Parasitemia peak (×10^4^ par/mL) and (% parasite reduction)
Untreated^1^	-	150.98±27.3
Bz 50 mg/kg^1^	p.o.	9.6±10.2 (93%)*
DB766 50 mg/kg^1^	ip	0.9±1.1 (99.4%)*
DB766 50 mg/kg+Bz 50 mg/kg^1^	ip+p.o.	0.0±0.0 (100%)* ^ and ^ **
Untreated^2^	-	95±12.8
Bz 50 mg/kg^2^	p.o.	10.5±14.4 (89%)*
DB766 50 mg/kg^2^	p.o.	78±88 (17%)
DB766 50 mg/kg+Bz 50 mg/kg^2^	p.o.+p.o.	43.6±31 (54%)*
DB766 50 mg/kg^2^	ip	4±3 (96%)*
DB766 50 mg/kg+Bz 50 mg/kg^2^	ip+p.o.	0.5±1 (99.5%)* ^ and ^ **
DB289 25 mg/kg^2^	p.o.	26.4±14.4 (72%)*
DB289 25 mg/kg+Bz 50 mg/kg^2^	p.o.+p.o.	1.2±1 (99%)* ^ and ^ **

1 and 2 = two independent representative studies.

*The single asterisk indicates statistically significant differences (p≤0.05) between untreated and treated groups.

**The double asterisk indicates statistically significant differences (p≤0.05) between the animal group that received the compound alone and in combination with Bz.

**Figure 3:**
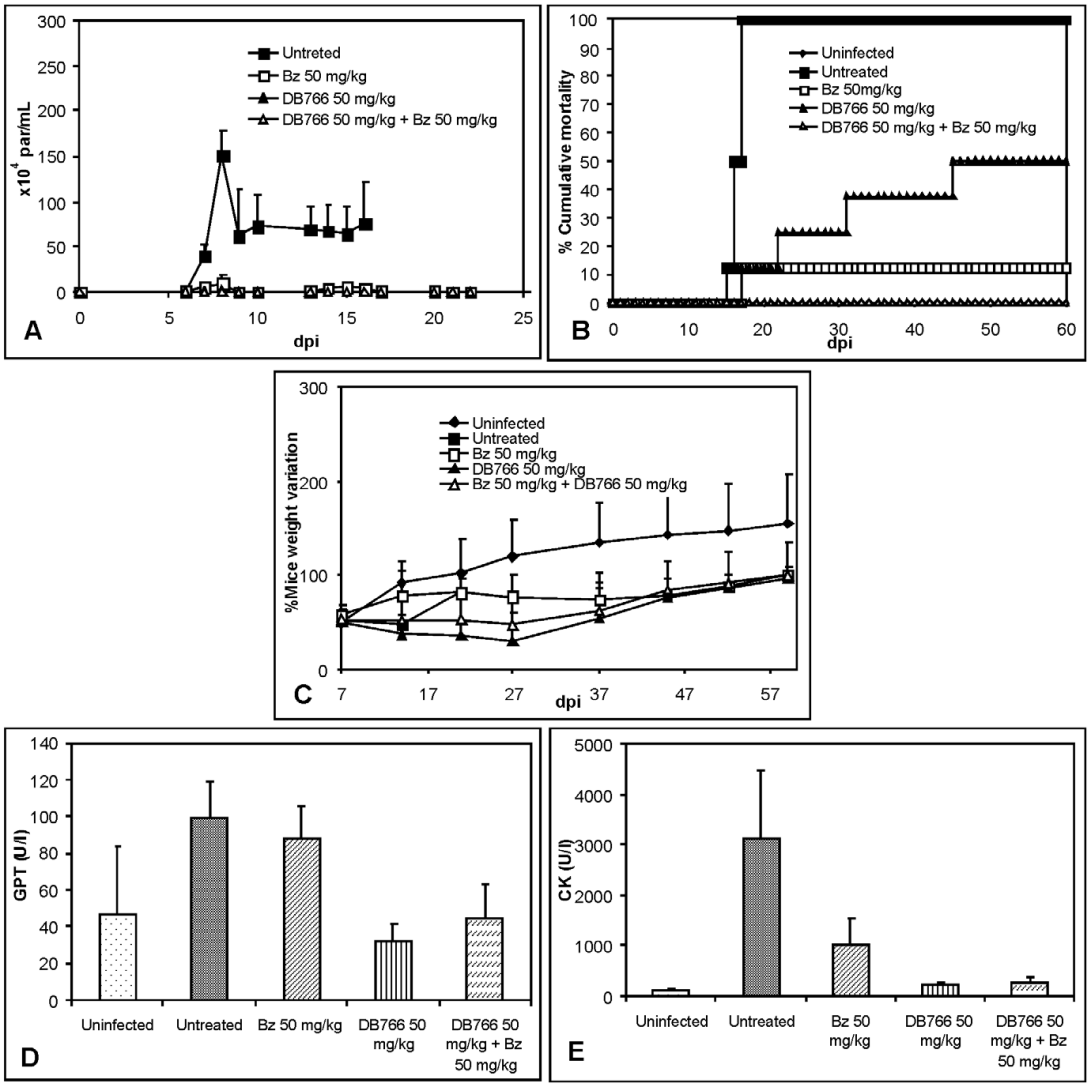
Activity of DB766 (ip.) combined or not with benznidazole (Bz) upon *T. cruzi*-infection in mice. (A): Parasitemia curve, (B) Cumulative Mortality and (D) Ponderal Curve of infected mice treated or not for twenty daily doses with 50 mg/kg/day DB766 combined or not with 50 mg/kg/day Bz. Plasma levels of (D) Glutamate pyruvate transaminase (GPT) and of (E) Creatinine kinase (CK) at 14 dpi from uninfected, untreated, DB766, Bz and combined therapy treated mice.

Since our recent study demonstrated the high efficacy of DB766 upon *T. cruzi* infection *in vivo* and *in vitro* [27], its combination with Bz was evaluated. Using the same therapy scheme as above, we found that the treatment of infected mice with 50 mg/kg/day of Bz or DB766 (ip.) or with DB766 (ip.) plus Bz (50 mg/kg/day each) resulted in decreases in mice parasitemia by about 90%, >96% and 100%, respectively, as compared to the untreated mice group (Fig. 3A, Table 2). The analysis of cumulative mortality revealed that while DB766 treated, Bz treated and untreated groups resulted in 50, 12.5 and 100% of death, the combined therapy of Bz plus DB766 resulted in 100% of protection, avoiding animal death (Fig. 3B). We found at 20 dpi, that the Bz treated and the combined therapy group showed partial restoration of the mice body weight compared to uninfected mice, however, the group treated with DB766 alone displayed even higher body weight loss than that of the untreated mice group (Fig. 3C).

In these experimental groups, at the 14 dpi, we found that the *T. cruzi* infection led to an increase in biochemical markers, resulting in a rise of 2- and 29-fold for GPT and CK levels, respectively (Fig. 3 D–E). Bz treatment alone did not reverse the hepatic damage induced by parasite infection [35], however, DB766 alone or in combination with Bz produced a decrease of about 69% and 57% in GPT levels when compared to untreated control (Fig. 3E). Muscle lesions, as evaluated by CK plasma levels were decreased with Bz treatment by about 70%. Treatment with DB766 alone or combined with Bz resulted in reductions greater than 91% when compared to untreated control (Fig. 3D). Histopathological assays confirmed the high efficacy of DB766 combined with Bz which resulted in 100% reduction in cardiac parasitism as compared to untreated animals (data not shown). Cure assessment also revealed that the administration of 50 mg/kg/day DB766 (ip.) plus 50 mg/kg/day Bz (p.o.) resulted in a 13% parasitological cure, as evaluated by both hemocultive and PCR analysis (Table 1).

**Figure 4:**
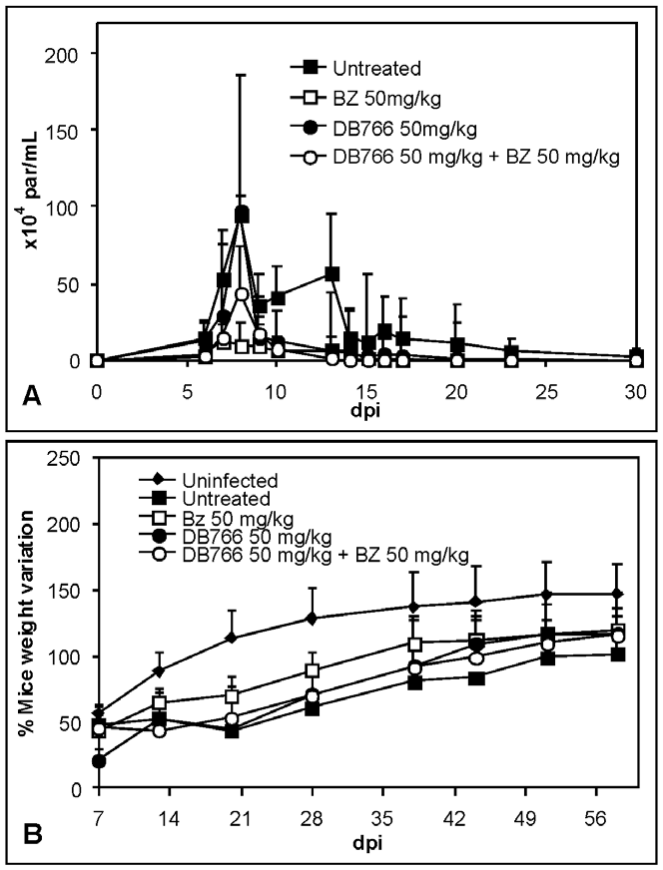
Activity of DB766 (p.o.) combined or not with benznidazole (Bz) upon *T. cruzi*-infection in mice. (A): Parasitemia curve, and (B) Ponderal Curve of infected mice treated or not for twenty daily doses with 50 mg/kg/day DB766 (per oral) combined or not with 50 mg/kg/day Bz.

Since DB766 shows high trypanocidal efficacy against *T. cruzi* in *vivo* on oral administration at 100 mg/kg dose [27], we evaluated another combination dosing regimen using sub-optimum oral doses of both DB766 (50 mg/kg/day by p.o.) and Bz (50 mg/kg/day by p.o.). DB766 (p.o.) alone reduced parasitemia by only about 20% (Fig. 4A) and the mortality rates were reduced by about 25% (data not shown). The administration of Bz plus DB766 (both p.o) decreased the parasitemia by 54% (Fig. 4A, Table 2), and gave a 100% mice survival rate similar to that of Bz alone (data not shown). At 20 dpi, both Bz alone and the combined therapy provide a partial recovery of mice body weight, however DB766 alone displayed similar body weight loss to that of untreated animals (Fig. 4B). In the oral treatment studies, the only parasitological cure noted by the hemoculture and PCR methods was for the group treated with 50 mg/kg/day DB766 (p.o.) (Table 1).

## Discussion

Since the introduction of Nifurtimox and Bz (1960–1970), despite the urgent need for new CD therapies, only allopurinol and a few azoles have moved to clinical trials, possibly due to (i) limited investment in this research field, (ii) an earlier mistaken concept that during the later phase of CD parasitism was absent and thus was not relevant to disease outcome and pathogenesis, and (iii) the absence of universal standardized protocols for drug screening [16]. The current profile for development of new drug candidates for CD includes 1) efficacy against different stocks, 2) efficacy against parasite forms relevant to the infection of mammalian hosts for both the acute and chronic phases, 3) oral administration in a minimum number of doses, 4) reduced toxicity, 5) yield high levels of tissue accumulation and long terminal half lives and 6) low costs [16], [39]–[40].

In this study using a murine model of *T. cruzi* acute infection, the trypanocidal efficacy of pafuramidine (DB289) and the arylimidamide DB766 either alone or in combination with benznidazole was evaluated over a relatively short period of treatment (20 days) employing both intraperitoneal and oral administration.

Diamidine-containing compounds such as pentamidine and furamidine are DNA minor groove binders with broad-spectrum activity upon different species of human and veterinary pathogens [20]. DB289 is the orally active prodrug of DB75 (furamidine) that exerts microbicidal effects upon different pathogens including *Trypanosoma brucei*, *Pneumocystis jiroveci* and *Plasmodium falciparum*, [41]–[45]. Interestingly, DB289 showed good oral efficacy in murine models of human African trypanosomes (HAT) suggesting that sufficient quantities are absorbed from the mouse gastrointestinal tract, delivering this dicationic molecule across the gut mucosa [43], [46]. Similar to the therapy of HAT [43], oral efficacy is a desirable feature for treatment of CD. However, in contrast to the studies performed with *T. brucei* [43], DB289 alone was not very effective against *T. cruzi*. This difference may be due to the fact that in contrast to the African trypanosomes, *T. cruzi* has intracellular stages living inside the cytoplasm of host cells, which represents an additional obstacle for drug access and delivery. We have found that the combination of Bz with DB289 improved the efficacy of the diamidine by reducing parasitemia and resulted in protection against mortality. In addition, this combined therapy provided a 9-fold enhancement of activity compared to that of Bz alone. Despite showing efficacy in Phase III clinical trials against HAT and initial indications of low toxicity in African, Asian, Caucasian and Hispanic populations, further studies with DB289 revealed considerable side effects leading to its withdrawal from advanced human trials [16], [43]. In fact, when higher doses of DB289 (≥100 mg/kg/day) were evaluated against *T. cruzi* infection *in vivo* (unpublished data of DGJB), higher numbers of circulating parasites and mortality rates were noticed as compared to untreated mice, perhaps a consequence of compound toxicity.

A previous study by our group demonstrated the beneficial effect of DB766 upon *T.cruzi in vivo*: a ten-day regimen of treatment reduced both blood and cardiac tissue parasitism, resulting in 90–100% protection against death even with an infection with naturally resistant *T. cruzi* strain (Colombian) to benznidazole [27]. Also, this AIA ameliorated electric heart alterations and reduced hepatic and heart lesions induced by the parasite infection [27]. Despite the promising trypanocidal effects of this AIA via ip (up to 50 mg/kg/day) and by p.o (100 mg/kg/day) routes which showed efficacy similar to Bz (100 mg/kg/day), DB766 (as well as Bz) failed to provide a parasitological cure [27]. This result may be a consequence of the highly stringent protocol employed (maximum of 10 days of drug administration). In fact, previous studies performed in *T. cruzi*-infected murine models with Bz and azoles reported high rates of parasitological cure only when dosing was continued for longer periods (≥40 days) [47]–[49], which supports using longer periods of therapy for our combination studies.

Our studies show that AIA are more active against *T.cruzi* than diamidine compounds [26], [50]. The greater activity may be related to differences in their physical properties since AD are highly basic molecules with pK_a_ values near 11 while AIA pK_a_ values are near 7. At physiological pH, AD are protonated and thus cationic molecules while AIA are essentially neutral molecules enabling their passive diffusion through the plasma membranes of both parasites and host cells. This large difference in properties likely affects absorption and distribution and may play an important role in the different activity of these two classes of compounds.

Our data showed that while DB766 alone reduced parasitemia giving a superior result to that of Bz, the combination of Bz and DB766 leads to undetectable parasitism, thus improving the efficacy of both compounds, especially Bz, whose potency was increased at least 20-fold. The improved activity of the combined therapy may reflect different targets (still incompletely understood for both) and/or effects upon different parasite forms. As reported, intracellular parasites must be considered the main parasite stage for drug targeting in CD since *T. cruzi is* an obligatory intracellular parasite [28].

As previously reported, DB766 displayed oral efficacy against an experimental *T. cruzi* infection when high but non-toxic doses (100 mg/kg/day) are employed [27]. However, when we evaluated the p.o. treatment with Bz and DB766 using sub-effective doses of both compounds (50 mg/kg/day each), the combined therapy only enhanced the activity of the AIA by 1.8 fold. The combined therapy showed a lower effect on parasitemia (but not on mortality rates) as compared to Bz treatment alone, suggesting an antagonistic effect that deserves to be further explored. One out of three surviving mice treated with DB766 by p.o. was cured as assessed by both hemocultive and PCR analysis. Although we did not find a considerable reduction in the mean parasitemia in this mice group, the cured animal was the one that displayed the lowest level of circulating parasitism, reaching undetectable parasitism (by light microscopy counting) after 23 dpi.

Although no visible adverse effects were noticed for DB289 and DB766, when they were used alone, both increased the cachexia induced by the parasite infection. This effect raises the possibility of drug toxicity and/or up-regulation of inflammatory mediator levels such as TNF-alpha that is implicated in loss of mice weight during *T. cruzi* acute infection [51]. Although no detectable acute toxicity was observed in mice treated up to a cumulative dose of 200 mg/kg/day of DB766 [27] and 100 mg/kg/day DB289 [43], and our data showed reduced hepatic and muscle lesions during the treatment of infected mice with DB766, a detailed biochemical and histopathological analysis is needed to clarify this matter.

The measurement of pro and anti-inflammatory cytokines in the plasma of infected and treated mice would contribute to the understanding of the possible role of these mediators upon drug toxicity and efficacy. Although no data is available for AIA, some studies suggest a regulatory effect by pentamidine upon pro-inflammatory cytokines [52]–[53]. Additionally, Bz down-regulates the synthesis of TNF-alpha by murine stimulated macrophages [54], ameliorates LPS-induced inflammatory response in mice by decreasing peak levels of this serum cytokine [55] and markedly reduces the production of pro-inflammatory cytokines and NO-derived metabolites in experimentally *T. cruzi*-infected rats [56]. These data may explain the weight recovery found in Bz-infected treated mice as compared to untreated mice since this pro-inflammatory mediator is strongly expressed in *T. cruzi*-infected mice [56]–[57], and is thought to be related to mice weight loss [51]. In our studies, we found a correlation between mice cachexia and mortality rates, including in the DB766 groups (ip. and p.o.) that may explain the lower protection against animal mortality in the animal groups that only received the AIA.

In conclusion, this study has shown that DB766 is much more potent in this mouse experimental model of *T. cruzi* infection than DB289 and that the trypanocidal activity is improved by combination therapy of both AD and AIA with Bz. Our data support additional studies with other diamidines and AIAs alone or in combination with other drugs with the goal of identification of new candidate therapies for the treatment of Chagas disease.
